# The Role of Zinc(II) Ion in Fluorescence Tuning of Tridentate Pincers: A Review

**DOI:** 10.3390/molecules25214984

**Published:** 2020-10-28

**Authors:** Rosita Diana, Barbara Panunzi

**Affiliations:** Department of Agriculture, University of Napoli Federico II, via Università 100, 80055 Portici NA, Italy; rosita.diana@unina.it

**Keywords:** zinc ion, fluorescence, tridentate ligand

## Abstract

Tridentate ligands are simple low-cost pincers, easy to synthetize, and able to guarantee stability to the derived complexes. On the other hand, due to its unique mix of structural and optical properties, zinc(II) ion is an excellent candidate to modulate the emission pattern as desired. The present work is an overview of selected articles about zinc(II) complexes showing a tuned fluorescence response with respect to their tridentate ligands. A classification of the tridentate pincers was carried out according to the binding donor atom groups, specifically nitrogen, oxygen, and sulfur donor atoms, and depending on the structure obtained upon coordination. Fluorescence properties of the ligands and the related complexes were compared and discussed both in solution and in the solid state, keeping an eye on possible applications.

## 1. Introduction

Over the past 20 years, fluorescence-responsive compounds are increasingly required for many technological applications, from lighting and switch devices to bio-imaging and analytical probes. Materials based on transition metal complexes were advantageously utilized. In this area, interest is growing in the abundant, less expensive, and environmentally “green” zinc(II) metal cation. Today, science is in great demand to address the challenge of sustainability. Scientific innovations and advances must play a major role in technological breakthroughs thanks to the choice of sustainable green matter as a substitute for highly toxic, expensive, and difficult to dispose products. So, green chemistry as the design of less hazardous chemical products and processes is a hot topic today. The replacement of heavy metal atoms in aromatic macrostructures with the small eco-friendly zinc cation, able to modulate the properties of coordination environments, easier to synthetize, and low cost, can be a way to meet the challenge.

From a research point of view, to the advantage of a large variety of coordination geometries and elaborate molecular architectures, zinc(II) complexes add the versatility of the luminescent levels, both in solution and in the solid state. Real breakthroughs for the novel luminescent technologies were obtained by employing highly efficient, stable, and cheap emitters. Among them, several zinc(II) complexes have to be included [1,2,3,4,5,6].

In the coordination complexes, electronic charge can be transferred between different molecular entities in the complex, from the electron donor zone to the receiving electron acceptor zone. In most complexes, charge-transfer electronic bands involve electron transfer between metal atoms and ligands. The charge-transfer bands in transition metal complexes are due to the shift of charge density between orbitals predominantly metal in character and those predominantly ligand in character. Most transitions are ligand-to-metal charge-transfer (LMCT) if the transfer occurs from the ligand orbitals to the metal, or metal-to-ligand charge-transfer (MLCT) in the reverse case. Due to its d^10^ closed shell configuration, zinc(II) ion has no optical signature. The d–d electronic transitions are not expected in zinc emissive complexes and the lowest energy excited states are mainly of a ligand-centered charge transfer (LCT) nature (as intramolecular charge transfer, ICT, and intraligand charge transfer, ILCT) and/or ligand-to-ligand charge transfer (LLCT) nature, rarely due to ligand-to-metal charge transfer (LMCT) states involving s or p empty orbitals of the metal [7]. The absence of ligand field stabilization energy leads to the formation of complexes with various coordination numbers, such as 4, 5, or 6, with tetrahedral, square pyramidal/trigonal bipyramidal, and octahedral geometries.

In dependence of the type of ligands and the coordination pattern imposed by the ligands, zinc(II) complexes exhibit fluorescence tuning both in intensity and/or emission maximum. Specifically, fluorescence enhancement (chelation enhanced fluorescence, CHEF mechanism [8,9,10,11]) or fluorescence reduction (metal-binding-induced fluorescence quenching [12]) can occur upon coordination. In addition, due to the lowering of the excited state of the bonded ligand upon coordination, a qualitative fluorescence tuning (blue or red shift of the emission maximum from ligand to metal) can be observed. The two different mechanisms can activate in solution and/or in the solid state, with drastic variations in fluorescence with respect to the ligand. Recently [13], the ability of the fluorophore ligand to form a π-contact with the metal cation was correlated with the fluorescence quenching or enhancement ability. Therefore, the information about the excited state geometry and the frontier orbital arrangement of the excited states is essential to understanding and foresight of the phenomenon.

The fluorescence enhancement effect is often the result of a stabilization of the excited state in poorly emissive ligands upon coordination [14]. Zinc(II) cation often causes a CHEF effect. Typically, in emissive zinc(II) complexes, the fluorescence emission results from a π-π* LCT and the role of the zinc ion is to freeze the favorable re-emissive conformation. In this way, it is possible to obtain strongly emissive materials for lighting devices by increasing the emission of organic molecules upon zinc coordination. Ligands with flexible spacers and appropriate aromatic moieties able to fold over the metal in a locked conformation show good potential in analytical and bio-chemistry. Many sensing systems for zinc cation detection exploit the CHEF effect [15,16,17]. At the opposite, fluorescence quenching is quite unusual in zinc complexes. Nevertheless, ICT or ILCT transitions can be responsible of zinc binding-induced fluorescence quenching. Fluorescence quenching is observed in rigid pyridine-based ligands upon coordination, due to a decreased HOMO–LUMO energy gap, or in zinc-induced quenching of the protein intrinsic fluorescence due to conformational perturbations [18,19,20,21].

Finally, the aggregate nature of materials consisting of fluorophores frozen into polymeric chains or networks give rise to noticeable changes in the energetic levels of the ligands. The assembly of emissive pincers by zinc coordination produces the most varied polymeric structures: coordination polymers (CPs) obtained by zinc bridges [22,23,24,25], metallated polymers obtained by coordination with pre-formed chains [26,27], and polymeric networks obtained by interlacing of flexible zinc-crossed fluorophores [28,29,30]. Owing to both the restrictions imposed to fluorophore and the efficient electron hopping in the tight structure, relevant emission tuning with respect to the free ligands and to mononuclear structures is envisaged.

As modifications of the ligand are expected to change energy levels and structural features, the number of binding sites in the pincer ligand plays a decisive role on the spectroscopic properties. Tridentate ligands are quite simple pincers, often low cost and easy to synthetize. At the same time, tridentate ligands guarantee good stability to the complex thanks to a relevant chelate effect. Ligands that can bind zinc(II) through three donor atom groups, such as *O*, *N*, and *S* donor atom groups, represent an interesting class due to the variety of behavior and applications. Tridentate zinc complexes are known to produce simple and mononuclear or intricate even polymeric structures, by themselves or with auxiliary ligands. By doing so, they can cause relevant fluorescence tuning with respect to the free ligand, in solution and/or in the solid state. In addition, depending on the charge of the pincer ligand, the zinc-binding reaction leaves unoccupied coordination sites for additional ligands, which in turn can modulate the structure and properties of the derived complexes.

Some representative functional groups involved in the build of fluorescence-responsive tridentate pincers can be identified. The Schiff base moiety, obtained by reaction of amines and carbonyl-containing compounds, is a good candidate for the synthesis of *N*, *O*, *S* donors containing ligands. In particular, in half-*salen*-type ligands, one nitrogen atom and one oxygen atom group chelate the metal, while the C=N functional group constitutes a versatile bridge between the *N*,*O* pincer and the branch of the ligand containing the third binding site. This role could be played by a third nitrogen, oxygen, or sulfur atom, as an example the carbonyl oxygen, the thione sulfur, or the donor atom of an heteroaromatic ring. The formation of a coordination core consisting of five- and/or six-membered rings between the pincer and zinc cation produces stable and sometimes highly emissive coordination complexes. Finally, the coordination core can be all made up of aromatic rings with *N*, *O*, *S* donor heteroatoms fixed in a rigid pincer or able to fold up as a flexible pincer.

This is an overview of selected cutting-edge examples of tridentate zinc(II) complexes causing fluorescence tuning with respect to the ligand. In this research area, many articles have been produced in the last 15 years. Starting from the synthetic and purely phenomenological approach, to the complete and detailed analysis of the energy levels, until the synergistic approach to the structure–property relationship, there is much to tell on this subject.

**Figure 1:**
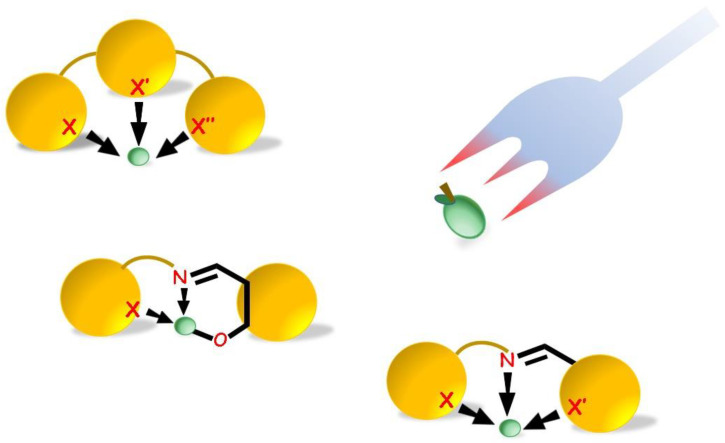
Schematic representation of most of the coordination cores achievable from tridentate pincers binding zinc(II) cation (in green). X, X′, and X″ can be *N*, *O*, and *S* atom groups.

In the following sections, ligands will be classified according to the binding donor atom groups, and fluorescence properties of ligands and related complexes will be comparatively discussed. By use of an easy “cartoon” representation (as in Figure 1), we will propose a quick intuitive overview of groups of structures, examined according to the photoluminescence (PL) response and the application area.

## 2. Nitrogen Binding Sites

The study of polydentate ligands with available *N* donor sites is a prolific research area in coordination chemistry [31,32,33,34,35,36,37,38,39,40,41,42,43]. Typically, nitrogen binding sites are neutral sites where the lone pair of nitrogen atom is available for donation. Nitrogen aromatic heterocycles are excellent building blocks for the synthesis of *N*-donor polydentate ligands. Single or fused five- and/or six-term rings produce stable and soluble pincers able to direct the chemical and chemo-physical properties. A substantial amount of literature articles on pyridine-containing tridentate ligands has emerged. Related to the enhanced π-electron delocalization upon zinc coordination, in some cases, the zinc-binding-induced fluorescence quenching phenomenon was detected. At the opposite, pyridine and other nitrogen heterocycles assembled in flexible architectures often produce a CHEF effect.

### 2.1. Terpyridine-Type Ligands

**Figure 2:**
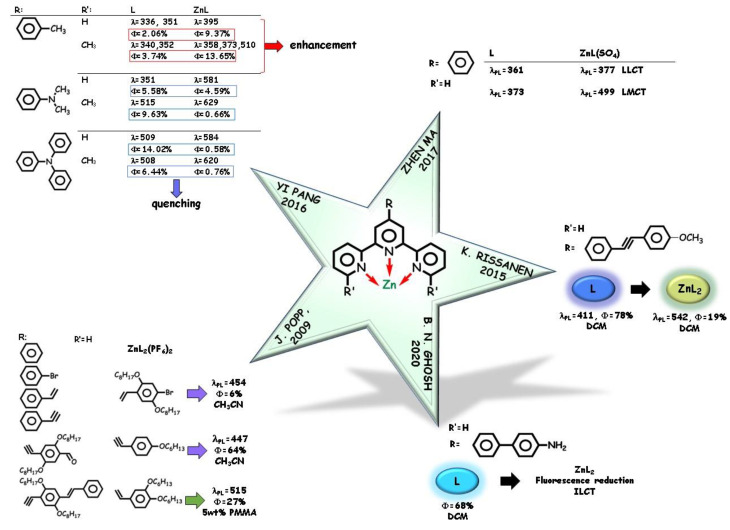
Tpy-type tridentate pincers causing zinc-binding fluorescence reduction/quenching.

Rigid pyridine-based tridentate ligands as terpyridine (Tpy) are well-known chelate ligands for transition metals [44,45,46]. Many Tpy ligands were found to be emissive in the solid state, with photoluminescence quantum yields (PLQYs) strongly depending on their own molecular structure and on substituents, with a relevant emission tuning due to metal coordination. Zinc-binding-induced fluorescence reduction/quenching was often detected, as well strong color emission tuning. In 2009, a series of zinc(II) bis(4′- phenyl-terpyridine) complexes with substituted Tpy were studied by J. Popp and coworkers [47] (Figure 2). The zinc ion is coordinated with the three nitrogen atoms from each of two terpyridine ligands and with two PF_6_^−^ as auxiliary ligands. Tuning of the color emission from violet to cyan (425–487 nm) in dependence of the extension of the π-conjugated system of the ligand was observed, and from low to high PLQYs (from 6 to 64%) were recorded. Thin solid films obtained by low-concentration dye-doped poly(methylmethacrylate) (PMMA) matrix show bright emission, with PLQYs up to 0.30. By DFT study, the HOMO energy level was also significantly influenced by the ligand structure, whereas the LUMO energy appeared to be independent of the electronic pattern of the Tpy ligand, with the enhanced π-electron delocalization leading to a decreased HOMO–LUMO energy gap.

The crystalline structure of a Tpy-type ligand upon coordination with different transition metals was examined in 2015 by K. Rissanen and coworkers [48] (Figure 2). The ligand displayed bright blue emission and high PLQYs dissolved in several organic solvents. A distorted octahedral arrangement with two tridentate terpyridine ligands was detected in the zinc complex and a significant greenish-yellow tuned emission attributed to ILCT states, still appreciable but lowered in intensity with respect to the ligand. DFT analysis rationalized the ICT-type electronic transitions involved from the diphenylacetylene moiety to the terpyridine group. Except for the other d^10^ closed shell cadmium (II) ion, a complete metal-binding-induced fluorescence quenching was observed in the presence of the other divalent metal ions.

Tpy-type ligands with different substituents were examined for their binding ability specifically toward zinc(II) cation in 2016 [20] and in 2017 [21] (Figure 2). In [21], Zhen Ma and coworkers examined the compound obtained by reacting 4′-phenyl-terpyridine and ZnSO_4_·7H_2_O by X-ray crystallographic analysis. A neutral 1:1 (Zn:Tpy) complex with a coordinated sulfate group, qualitatively emissive in the solid state, was obtained. The emission spectrum displayed two bands at ca. 377 and 499 nm red-shifted with respect to 361 and 373 nm for the ligand in the solid state. The former higher energy band of the complex was assigned to LLCT whereas the latter band to LMCT [49,50]. In 2016, the influence of the coordination stoichiometry was explored by Yi Pang and coworkers [20], by reacting substituted Tpy-type ligands (R in Figure 2 is a *p*-substituted phenyl ring bearing a donor group and R’=H or R’=CH_3_ on the lateral pyridine groups) with ZnCl_2_. The authors pointed out that the zinc complex forms in a 2:1 (ligand: metal) ratio with a low zinc(II) concentration and in a 1:1 ratio with a high concentration of metal. In ethanol, fluorescence quenching occurs in 2:1 complexes, whereas, turning into 1:1 complexes, fluorescence increases in the opposite direction, marking the role of the coordination pattern in the emission intensity. In 1:1 complexes, temperature-dependent fluorescence spectroscopy elucidated the role of the ICT mechanism between donor and acceptor groups. Due to the occurrence of a relevant ICT process, the strong donor substituents induce zinc-binding fluorescence quenching and red-shift of the emission maximum. Conversely, the weak donor substituent *p*-CH_3_-phenyl group causes an increase in the fluorescence emission of the 1:1 complex.

Very recently [51], B.N. Gosh and coworkers examined the X-ray crystal structure of 4′-functionalized terpyridine complexes (Figure 2). Zinc cation did show a trans-arrangement of the terminal pyridine nitrogen atoms with respect to the central pyridine ring in the 2:1 ligand zinc tridentate complex. The terpyridine ligand exhibits a bright blue emission in dichloromethane with 68% PLQY and produces a different coordination pattern in dependence of the coordinated metal. Upon zinc complexation, ligand emission shows a significant reduction while other transition metal ions completely quench the fluorescence of the ligand. The weak fluorescence of the zinc complex can be imputed as an ILCT transition from the amine moiety to the metal coordinated terpyridine fragment [48,52,53]. The non-fluorescence nature of the complexes with other metal cations can be imputed as MLCT-type transitions.

Recently, fluorogens exhibiting aggregation-induced emissive properties (AIEgens) have grabbed scholars’ attention in various scientific areas. In contrast to conventional aggregation-caused quenching (ACQ) molecules, AIEgens are weakly fluorescent/non-emissive in diluted solution and emit intensely in their aggregate form (concentrated solution and solid state), owing to the restriction of intramolecular motions [54]. Tpy-based zinc complexes AIEgens have been proved to be attractive and versatile tools for biological imaging and chemical sensing. Under physiological conditions, Tpy-type ligands are employed in bio-medical applications, such as for living cell imaging. As an example, in 2005 [55], Valery N. Kozhevnikov and Burkhard König presented 2,2′-bi- and 2,2′:6′,2″-terpyridines with aminomethyl and aryl substituents employable as luminescent probes coordinating in a pentadentate coordination core zinc cation in physiological media.

**Figure 3:**
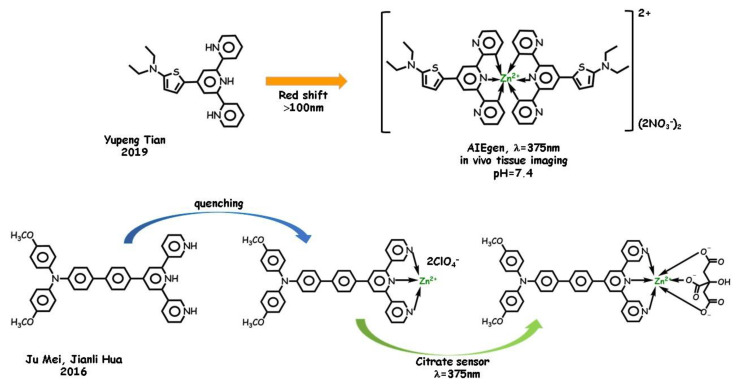
AIE behavior of zinc complexes from Tpy-type tridentate pincers.

In 2019, a thiophene bridged Tpy tridentate Zn(II) complex (Figure 3) was designed for RNA-specific targeting thanks to its AIE bright yellow-green fluorescence emission under physiological conditions by Ju Yupeng Tian, Dandan Li, and coworkers [56]. On the other hand, in 2016, a new fluorescence sensor for citrate (acting itself as a tridentate ligand) detection was developed by integrating an AIE Tpy ligand with zinc(II) by Ju Mei, Jianli Hua, and coworkers [57]. The enhanced electron-withdrawing ability of the complex gave rise to a red-shifted fluorescence compared with the organic ligand. The zinc-based probe was not an AIEgen but showed significant fluorescence enhancement (fluorescence turn-on mechanism) under substitution of the auxiliary ligands with citrate. The bathochromic shift of the absorption maximum and the decrease of fluorescence intensity may be ascribed to the stronger ICT in the complex as compared to the ligand (Figure 3).

### 2.2. N,N,N Schiff Base-Type Ligands

**Figure 4:**
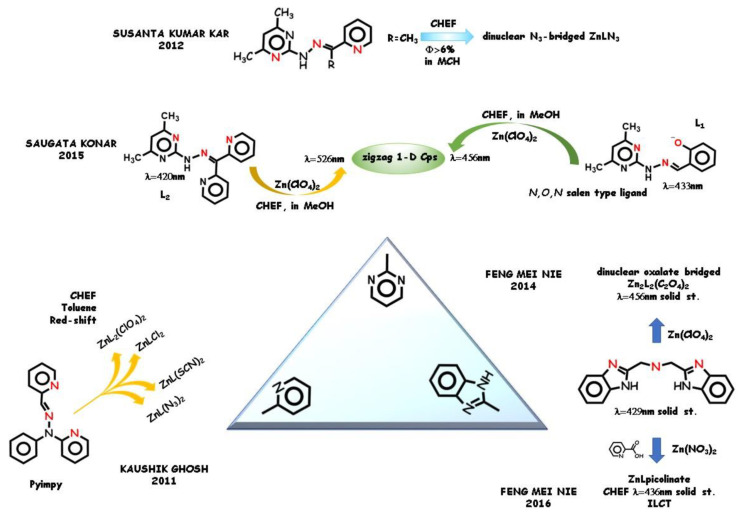
*N*,*N*,*N* Schiff base-type ligands with different nitrogen aromatic rings and their CHEF active complexes.

Schiff base ligands containing an additional nitrogen binding site, usually derived from a nitrogen heterocycle, have been widely reported. The versatile CH=N bridge allows the employment of a large variety of substituents and to build the most varied architectures. Unlike their complexes, this kind of ligand is often non-emissive, and a general behavior due to the CHEF effect was mostly found. In 2011, Kaushik Ghosh and coworkers [58] explored the crystal structure and photophysical properties of four zinc(II) complexes derived from the tridentate ligand Pyimpy (see Figure 4) with different auxiliary ligands. In the complexes, two pyridinic nitrogen groups are involved in the equatorial plane binding along with the iminic nitrogen group. The complexes show various fluorescence emission in toluene solution upon excitation of the charge transfer band near 350 nm (ascribable to π,π* LC of the ligand [59]) while the free ligand Pyimpy displays no fluorescence emission in the same experimental conditions. The phenomenon was ascribed to the loss of vibrational energy decay due to ligand stiffening under coordination. In addition to the zinc-binding fluorescence intensity enhancement, a shift of the maximum of emission was detected, in dependence of the auxiliary ligands.

Other nitrogen aromatic heterocycles have recently emerged as electron donor-containing moieties. Among them, pyrimidine groups attract attention for their role in biological systems [60,61]. Susanta Kumar Kar and coworkers in 2012 [62] prepared two tridentate *N*,*N*,*N* donor Schiff base ligands [63] using pyrimidine- and pyridine-containing carbonyl compounds (see Figure 4). Whereas the ligand with a methyl substituent was fluorescent silent, its zinc complex showed a strong CHEF effect in methylcyclohexane. Probably due to photoinduced electron transfer processes in the presence of several nonbonding electron pairs on the nitrogen donor atom groups, the ligand π,π* transitions are not allowed and the flexible bonds of the ligands cause the activation of the non-radiative channel. CHEF activates by the increase in conformational rigidity of the ligands upon strong zinc binding, which prevents non-radiative channels [64]. Pyrimidine-containing Schiff base ligands were studied in 2015 by Saugata Konar [65] for the zinc-binding ability into 1-D coordination polymers held together by μ_1,5_-bridged dicyanamide ions. The difference due to the activation of a *N*,*N*,*O* bonding site (ligand L1, achieved by the presence of a half-*salen* group) with respect to the *N*,*N*,*N* bonding site (ligand L2, see Figure 4) was pointed out. Both the ligands display low fluorescence intensity in methanol. Conversely, both their polymeric complexes show red-shifted enhanced fluorescence emission. The low emission intensity of the ligands was ascribed to photo-induced electron transfer processes, while the CHEF effect to an increase in conformational rigidity of the ligands upon complexation. The complex derived from the *N*,*N*,*O* ligand shows the highest CHEF effect compared to the complex derived from the *N*,*N*,*N* ligand. This has been attributed to the strong binding of the *salen*-type ligand L1 thanks to the *N*,*O* donor pincer compared to the *N*,*N* pincer of ligand L2. In the first case, the conformation of the coordination core is strongly trapped in a planar conjugated *habitus*.

Polydentate flexible ligands containing two or more benzimidazole donor units have long been used in coordination and supramolecular chemistry [66,67,68]. The fused benzimidazole ring contains the *N*-binding site of the imidazole moiety. Feng-Mei Nie and coworkers [69,70] synthetized solid-state emissive zinc(II) complexes derived from tridentate and polydentate benzimidazole (Figure 4), solid-state emissive by themselves. Different architectures were obtained in dependence from the number of chelating site and on the auxiliary ligands, as analyzed by the X-ray diffraction technique. In presence of oxalate, a dinuclear oxalate-bridges Zn_2_L_2_ coordination core was obtained (in 2014, [69]), whereas the same ligand produced a five-coordinate ZnL coordination core in the presence of *N*,*O* chelating picolinate (in 2016, [70]). In both cases, the fluorescence emission was assigned to π-π* ILCT bands. The red shift of the emission maxima from ligand to complexes is influenced by the coordination pattern. The remarkable fluorescence enhancement compared to the free ligand was ascribed to extensive π-conjugated structure formation.

### 2.3. Ligands for Sensing Analysis and for Supramolecular Architecture Building

Polydentate structures with flexible moieties are useful tools for the sensing of metal analytes by the fluorescence technique [71,72]. Many novel fluorescence chemosensors for zinc cations were recently explored. From a purely theoretical point of view, Hee-Seung Lee and coworkers in 2013 [14] explored the role of fluorophore−metal interaction in photoinduced electron transfer (PET) sensors and the large CHEF effect promoted by zinc(II) coordination by time-dependent density functional theory (TDDFT) study, pointing out how DFT study is the logical complement of the synthetical work about novel sensing molecules [17,30,59,73].

**Figure 5:**
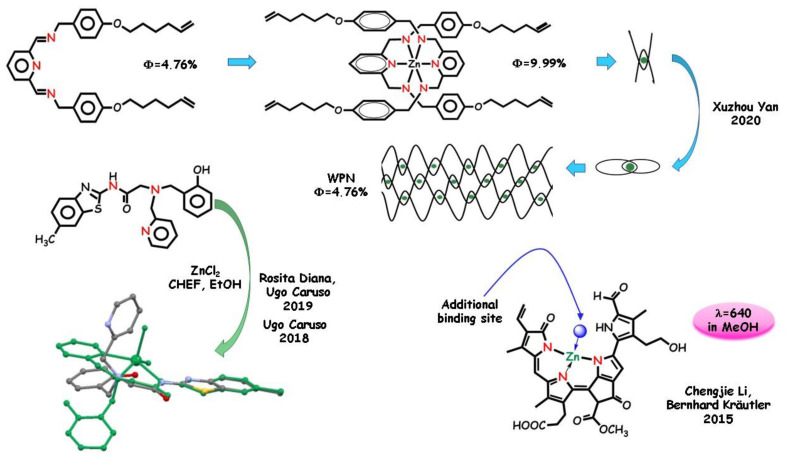
Supramolecular architectures produced from zinc-interlocked chains. Tridentate pincers for sensing analysis of zinc cations.

A significant example of a bendable *N*,*N*,*N* ligand useful as a sensor was synthetized and employed in 2018 by Ugo Caruso, Rosita Diana, and coworkers [25,59,74,75,76]. Specifically, the pyridine/phenol/benzoxazole-based ligand (Figure 5) able to bind various transition metals acts as *N*,*N*,*N* tridentate selective fluorogenic ligand toward zinc(II) by a sensing CHEF mechanism, in water or water/mixed solvents. DFT calculations for the free ligand and the complex were used to calculate frontier molecular orbitals. The frontier molecular orbitals undergo strong changes when the sensor folds back onto the metal cation (see Figure 5). HOMO of the ligand and of the complex are π orbitals with contributions from 2p orbitals of the carbon atoms in the benzothiazole ring. LUMO of the free ligand is a π* orbital with contributions mainly from the benzothiazole ring, while the LUMO of the complex is a π* orbital localized on the pyridine group. Not unusual for multidentate ligands, the same tripodal multidentate sensor acts as a tetradentate ligand toward zinc ion at pH = 8.0 [17]. In basic media, the sensor activates the phenate oxygen-binding site in addition to the *N*,*N*,*N* chelate site. The ligand results a pH-dependent sensor [77], able to detect zinc(II) ion in a neutral/slightly acidic and in a slightly basic aqueous environment with different emission responses.

Chlorophyll-catabolite named phyllobilins may display a capacity to complex metal ions. In 2015, in a mighty article [78], Chengjie Li and Bernhard Kräutler explored pink-colored phyllobiladienes as effective tridentate ligands, leaving one unoccupied coordination site that may be used for coordination by an external additional ligand, such as proteins or nucleobases (Figure 5). Coordination of the zinc cation to the scarcely luminescent pink chlorophyll catabolites induces bright fluorescence in the complex. The zinc(II) adduct ZnL shows strong red emission in solution (band picked around 650 nm, almost two orders of magnitude more intense than the free ligand) so it can be potentially used as in vivo sensors. Analysis of the fluorescence of MeOH solutions leads to quantitative detection of the cation thanks to the linear correlation between fluorescence intensity and zinc(II) concentrations.

*N*,*N*,*N* tridentate complexes have a part as novel polymeric materials with intriguing structural and mechanical features for the construction of smart supramolecular architectures. The formation of polymeric architectures through zinc cation linkers can be the way to increase and/or tune the fluorescence properties of the organic ligands, and to transfer the desired emission properties to macrostructures.

Mechanically interlocked molecules, such as catenanes [79,80,81], are topological structures held by mechanical bonds, with intriguing potential in several fields from synthetic chemistry to materials science and nanotechnology [82,83,84]. In 2020, Xuzhou Yan and coworkers [28] obtained a mononuclear ZnL_2_ complex by reacting a zinc salt with *N*,*N*,*N* chelating ring-like [2] catenane ligands. The synthesis of a “woven” polymer network (WPN) via ring-opening metathesis polymerization of the catenane produced a 3-D coordination polymer consisting of rigid metal-coordinated crossing points and flexible alkyl chain. The flexible and firm network obtained by interlaced fluorophore units exhibit different emission properties in the solid state with respect to the reagents. The mononuclear ZnL_2_ complex is an AIEgen, relatively flexible and less restricted. It can aggregate tightly in the solid state, resulting in a strong emission. After the formation of the more interlocked network structure, the restrictions imposed to fluorophore aggregation lower the emission. The quantum yields of the three structures (9.99% for ZnL_2_, 4.76% for the [2] catenane, and 8.97% for the WPN) measured in the solid state showed similar variation trends along with different topological structural transformations.

## 3. Nitrogen and Oxygen Binding Sites

*N*,*O* chelating Schiff bases ligands, often half-*salen*-type ligands, can be obtained by condensation of salicylaldehyde and its derivatives with a variety of primary amines. Applications of Schiff base complexes in various fields, such as molecular electronics, optical, catalysis, analytical, pharmaceutical, and biomedical [85,86,87,88,89,90,91,92,93,94,95,96,97,98,99,100], are known. The *salen* moiety owes attention to its versatility and coordination ability toward several metals as a mononegative ligand. Schiff bases ligands can form homo- and hetero-metallic complexes and 1-D, 2-D, and 3-D polymers. The emission behavior of many zinc(II) half-*salen* complexes has attracted interest due to their potential as light-emitting layers [101] and fluorescent sensors [102,103]. Photoluminescence properties of *N*,*O* Schiff base complexes can be changed/improved by the introduction of a third binding site at the ligand backbone. In this case, locking the metal in a strong *N*,*O* clamp, properties can be modulated by insertion of the third donor atom group in a suitable site of the binding architecture. Tuning of fluorescence emission is expected by varying the third donor atom and its position, by addition of substituents on the coordination core and by the auxiliary ligands. The most recent and intriguing advances in the design of *N*,*O*,*N* and *O*,*N*,*O* tridentate ligands for zinc(II) complexes are presented below.

### 3.1. N,N,O Ligands

Many *N*,*N*,*O* ligands have a relatively simple structure. By addition to the mononegative *N*,*O* half-*salen* block of an aromatic or non-aromatic nitrogen-containing fragment, a wide variety of structures can be obtained. In many articles, X-ray diffraction analysis constitutes the starting point to correlate structural data and theoretical analysis. On the other hand, more elaborate *N*,*N*,*O* chelating structures have been designed for specific functions. Because zinc is essential to life as part of enzymes, the detection of zinc cation in complex biological systems is a desirable goal. Moreover, zinc complexes have recently been employed as probes in fluorescence bio-imaging techniques.

#### 3.1.1. Half-*Salen*-Type Ligands

**Figure 6:**
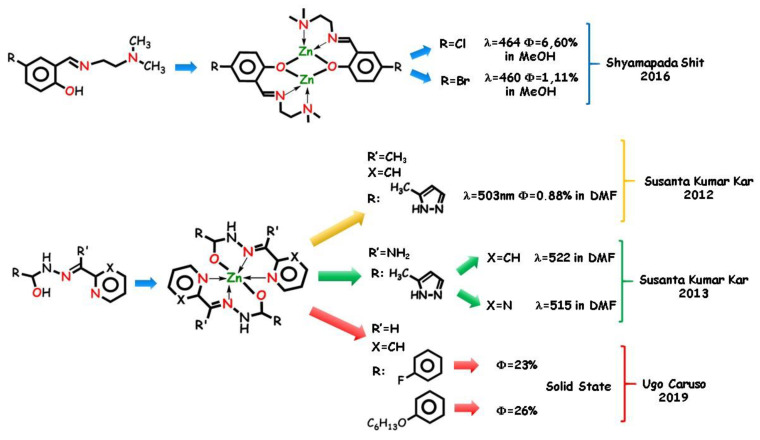
*N*,*N*,*O* pincers containing half-*salen* moiety and related complexes.

In 2016, Shyamapada Shit and coworkers [104] presented two tridentate *salen* Schiff base ligands with two different halogen substituents in combination with azide as auxiliary ligands (Figure 6). The structure–property relationships of the coordination complexes were examined. Single-crystal X-ray diffraction studies revealed a similar dinuclear pattern for the two complexes. Spectroscopic characterizations revealed that the fluorescence pattern of the complexes is scarcely affected by the halogen substituent, in both case distant from the coordination core and with no relevant electronic effect. As expected, the emission recorded in methanol, ascribable to ILCT of the complexes, is significantly higher than that of the corresponding ligands.

Variously substituted polydentate hydrazones are important scaffolds in coordination chemistry REFF. Tridentate *N*,*O*,*N* ligands can explain their chelating ability utilizing the pyridine/pyrazine *N* atom, one azomethine *N* atom, and one carbohydrazide *O* atom, and can mold itself according to the required coordination of the metal ion. Pyrazole-based flexible *N*,*N*,*O* hydrazones and their zinc(II) complexes were studied by Susanta Kumar Kar and coworkers in 2012 [63]. By the X-ray technique, the flexible pyridyl–pyrazolyl-ended ligand was found to be able to produce different coordination structures with different metal ions, and relevant changes in the luminescent pattern (Figure 6). In DMF, d^10^ ions, such as Zn(II) and Cd(II) cations, show a high CHEF effect, unlike the Ni(II) ion, which in turn causes fluorescence quenching with respect to the free ligand. In 2013 Kumer Kar and coworkers [105] observed no relevant fluorescence in the ligands, while its cadmium(II) and zinc(II) complexes were emissive in DMF, due to intraligand p,p* and n,p* transitions and also to the weak MLCT band [64]. Chelation-induced rigidity also plays an important role impeding the nonradiative channels due to the flexible bonds.

Recently, the interest in this class of complexes was promoted by the photoluminescence activity in the solid phase, as required for emitting layers of LEDs and solar cells. In 2019, Ugo Caruso and coworkers [75] obtained two complexes by reaction of zinc(II) acetate and *N*,*N*,*O* tridentate pyridinyl-hydrazone ligands (Figure 6). Both ligands have a pyridinyl-hydrazone moiety acting as mono-negative tridentate ligands toward the zinc ion in a 2:1 stoichiometric ratio, producing an octahedral environment. Ligands and complexes are scarcely emissive in diluted solution. The crystalline ligands show poor emission in the solid state while the *push-pull* more efficient pattern of the complexes guarantee intense solid-state blue fluorescence due to the AIE (aggregation-induced emission) effect [106]. In this case, the fluorescence pattern of the complexes is largely affected by the substituent. Quantum yields above 20% and large Stoke’s shifts were recorded. Because large Stoke’s shifts eliminate spectral overlap between absorption and emission phenomena, the detection of the fluorescence improves both in the intensity and in color purity, making the complexes promising for actual applications.

#### 3.1.2. Ligands for Sensing Analysis and for Biological Applications

**Figure 7:**
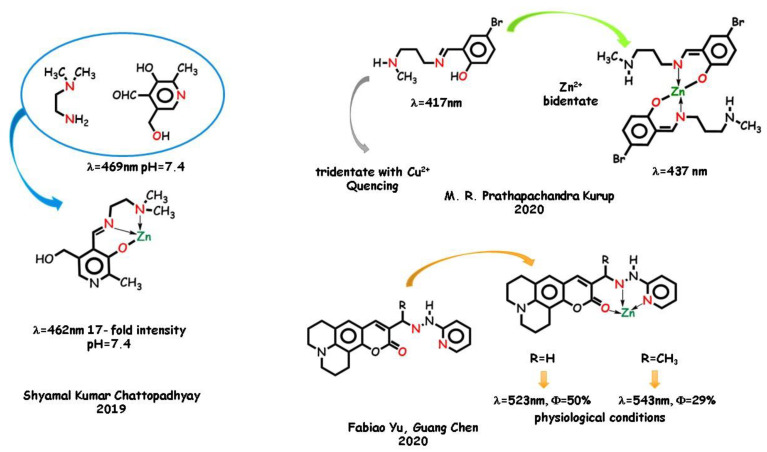
*N*,*N*,*O* pincers for sensing analysis and biological applications.

In order to achieve the goal of zinc(II) detection in complex biological systems, or to design zinc-based architectures with biological activity, the C=N moiety included in flexible ligands can be an easy synthetic solution. A half-*salen*-type *N*,*O*,*N* ligand [107] was prepared in situ by Shyamal Kumar Chattopadhyay in 2019 (Figure 7) and employed to produce a mononuclear zinc(II) complex whose structure was determined by single crystal X-ray diffraction. In an aqueous methanol solution at the physiological pH, the complex exhibits an intense greenish-blue fluorescence whose maximum does not differ substantially with respect to the free ligand while the intensity is about 17-fold stronger. The ability to give fluorescence in aqueous solutions makes the probe promising for DNA binding activity and fluorescence bio-imaging. By DFT calculations, the nature of the electronic transitions was assigned to the π,π* transitions of the imine and heterocyclic moiety and to a n,π* transition for the free ligand, and to π,π* LCT transition in the complex.

Designed for cytotoxic and antibacterial activity by M.R. Prathapachandra Kurup and coworkers in 2020 [108], a potentially *N*,*N*,*O* tridentate ligand worked with copper and zinc salts with unexpected different results. The two complexes (Figure 7) show a very different structural and spectroscopic pattern. In solution, the fluorescence emission intensity of the ligand decreased on complexation with Cu(II) ion, which can be attributed to the decrease in electron density on the ligand due to d orbital being involved. Contrarily, the ligand acts as a bidentate pincer toward zinc(II), coordinating to the metal cation through phenoxo oxygen and imine nitrogen. The bis-chelate metal complex produces an enhancement in the fluorescent intensity in DMF, due to the prevention of the photoinduced electron transfer process preserving ILCT bands.

Very recently [109], two *N*,*O*,*N* tridentate ligands were used by Fabiao Yu, Guang Chen, and coworkers as fluorescent sensors to monitor intracellular zinc(II) in living cells by fluorescent bioimaging. The elaborate fused-rings aromatic part guarantees a rich π-conjugated system able to give a photoluminescent response. The oxygen atom group of the C=O fragment is the third neutral jaw of the tridentate ligand. Due to the restriction of the isomerization and rotation of C=N upon coordination, the probes show fluorescence enhancement (from 4 to 7-fold at 523 and at 543 nm, respectively) and large Stokes shifts of the emission spectra. A real-time two-photon excitation wavelength apt to biological experiments and deep penetration in tissues was detected.

### 3.2. O,N,O Ligands

The ubiquitous Schiff base moiety is the useful fragment also in this case. Due to the versatile synthesis and the coordination ability of the CH=N functional group, many binegative ligands were built starting from *O*,*N* chelating *salen*-derivatives and a third oxygen or sulphur-containing moiety.

**Figure 8:**
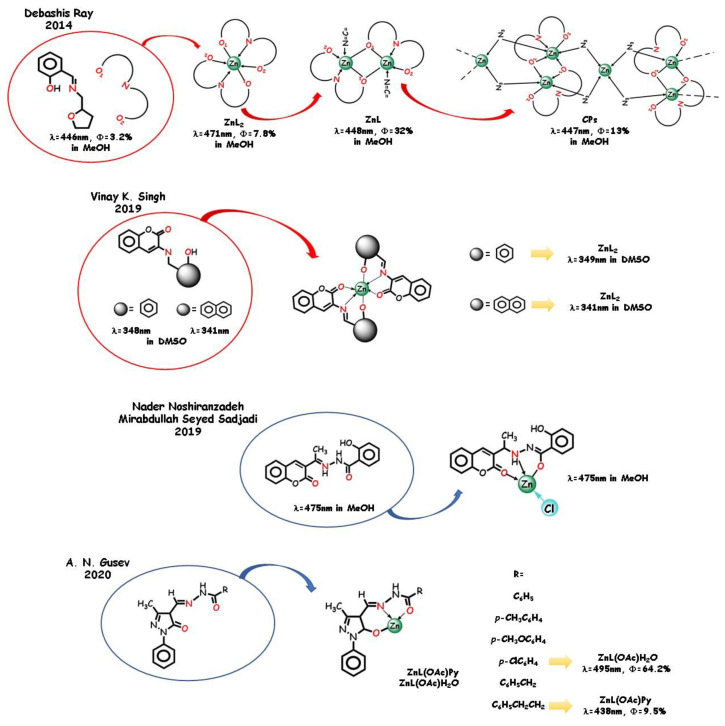
Selected examples of *O*,*N*,*O* ligands and zinc complexes containing the C=N moiety.

Tridentate furan-containing half-*salen*-type ligands were published by Debashis Ray and coworkers in 2014 [22] (Figure 8). Participation of the furan oxygen group in coordination is scarcely reported. The phenoxido-O group can be involved in coordination as a neutral donor site. The coordination abilities of the furan ring and the effect of several auxiliary triatomic bridging groups were checked by reacting zinc perchlorate salt in the absence and in the presence of auxiliary thiocyanato or azido anions. The ligand coordinates as a tridentate ligand producing a mononuclear specie, a dinuclear specie in the presence of thiocyanato, and a polymeric azido-bridged chain with azido anion. In MeOH solution, the emission bands of ligand and complexes are very similar. The PET process due to the presence of an electron lone pair of the donor atoms in the ligand produces a low PL quantum yield. Zinc-binding-induced emission greatly depends on the coordination pattern. The coordination-driven enhancement of fluorescence intensity is explainable with an increased rigidity upon complexation, so that the emission intensity in the dinuclear-bridged complex is higher than in the mono and polynuclear.

Coumarin-based molecules were recently employed as laser dyes and fluorescent probes [110,111,112]. Zinc‑selective coumarin-based chemosensors were used in biological systems. Vinay K. Singh and coworkers in 2019 [113] produced two mononegative *O*,*N*,*O* tridentate Schiff base ligands employed in the coordination of various metal cations (Figure 8). The structural information obtained by the X-ray technique was used in the structure–activity correlation. In contrast to the fluorescence quenching upon cobalt, nickel, and copper complexation, zinc complexes show a from medium to strong emission, due to the locally excited π*,n transition state, the nature of substituents, and the conformational rigidity of the fluorophore greatly affecting the photo-induced electron transfer processes. Another coumarine-containing tridentate ligand with a hydrazonic flexible skeleton was studied by Nader Noshiranzadeh and Mirabdullah Seyed Sadjadi in 2019 [114], focusing its catalytic activity in azide-nitrile cycloaddition reactions (Figure 8). The combination of the coumarin moiety and hydrazone functional group did show interesting optical properties. The ligand acts as a mononegative *O*,*N*,*O* tridentate trough the azomethine nitrogen and the esteric oxygen atom groups to the metal ion. Methanol and a chloride ion complete the coordination sphere. The ligand itself exhibits an intense fluorescent emission in methanol at 475 nm, which can be assigned to the p,p* transfers. Interestingly, as the ligand is encumbered, the nonradiative channels due to the flexible bonds are impeded and the fluorescence intensity is scarcely affected by zinc coordination. The higher emission band of the complex is very similar both in intensity and in the maximum wavelength, still related to intraligand emissions.

Very recently [115], a series of mononuclear acetate-containing zinc complexes derived from acylhydrazones demonstrated efficient photoluminescence in the solid state, with emission maxima from 414 to 536 nm and quantum yields from 9.5 to 64.2% depending on the nature of the acyl fragment and of the auxiliary ligand (water or pyridine). A.N. Gusev and coworkers in 2020 synthetized several hydrazones containing a phenylpyrazole fragment acting as mononegative ligands toward the cation by deprotonation of the pyrazole fragment. The ligands themselves are poor emitters in the solid state. The PL efficiency of the hydrate complexes is lower with respect to the pyridinium analogs in the case of the aromatic acid derivatives, whereas an inverse dependence was observed for the phenylalkyl derivatives.

**Figure 9:**
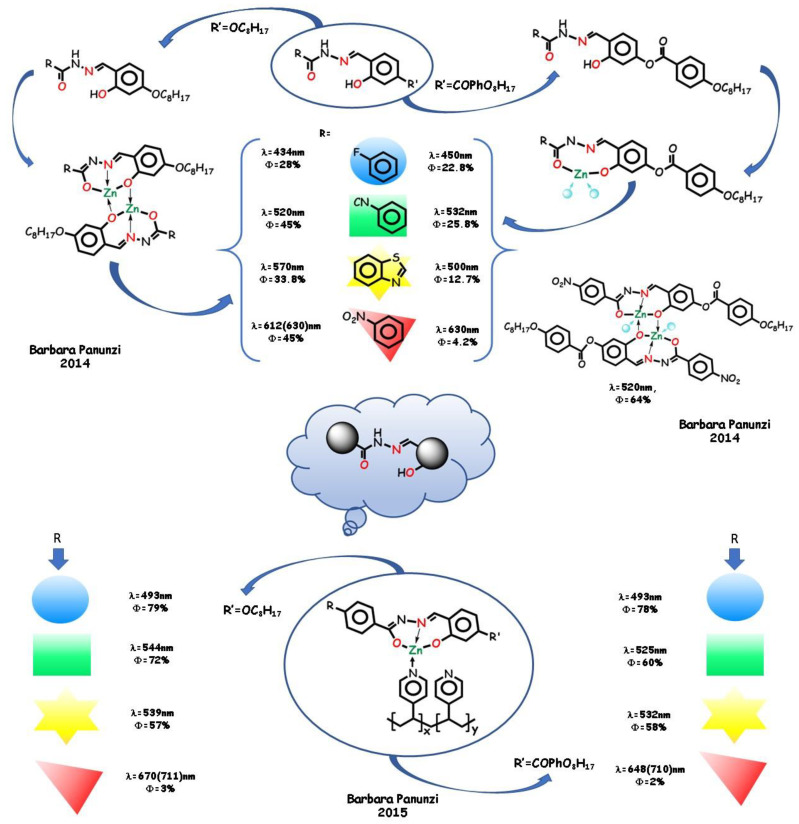
Aroyl- and acylhydrazones *N*,*O*,*N* tridentate pincers with different substituents, the derived complexes, and the related zinc polymers.

A systematic approach based on zinc-binding aroyl- and acylhydrazones ligands with different substituents and pyridine rings as auxiliary ligands was adopted in a series of articles by B. Panunzi and coworkers (Figure 9). This approach, based on the study of a homogeneous set of the same skeleton ligands, which differ in one relevant substituent, led to highly stable mononuclear and polynuclear structures and to metallated zinc polymers emissive in the solid state.

In 2014, B. Panunzi and coworkers reported the synthesis and characterization of four *O*,*N*,*O* acylhydrazono [23] and four analogous aroylhydrazono-type [116] ligands. The difference within the first mononuclear complex group and within the second (mono and dinuclear) groups is the electron-acceptor substituent R on the same tridentate chelating core. The difference between the two groups is an additional benzyloxy bulky group (Figure 9). In both cases, the enhanced fluorescence in the solid state is due to the increased rigidity upon coordination, which leads to a decreased probability of electronic nonradiative transitions from the excited states [117,118]. Tuning of the emission wavelength was achievable by varying the electron-acceptor group R with significant analogies in the two series (see Figure 9). DFT analysis produced a first rationalization of the red shift in the chromophore series. The ability of pyridine molecules to complete the coordination sphere of zinc(II) was explored and its dominant contribution to LUMO involved in the electronic transitions was pointed up. Due to self-quenching decreasing, the photoluminescence intensity enhances by increasing the distance between the emitting species in the crystalline complexes. Therefore, the second series of bulky complexes show higher PLQYs with respect to the first series. An unprecedented 64% PLQY for the R=CN bulky zinc complex was recorded, with relevant tuning in the wavelength and emission intensity with respect to the ligand; this value is suitable for lighting applications.

In order to transfer the optimal fluorescence performance of the two groups of complexes into polymeric materials, the same tridentate ligands were employed by B. Panunzi and coworkers in 2015 to prepare metallopolymers by chemical grafting of Zn(II) coordinating cores onto preformed poly(4-vinylpyridine) (PVPy) chains [119] (Figure 9). As an alternative approach to the dye-doped materials, this practice showed advantages, such as stability of the materials, synthetic easiness, and reproducibility. In the 10 wt.% grafted polymeric materials, effective emission color tuning was achieved depending on the strength of the electron acceptor substituent and high solid-state PLQYs.

**Figure 10:**
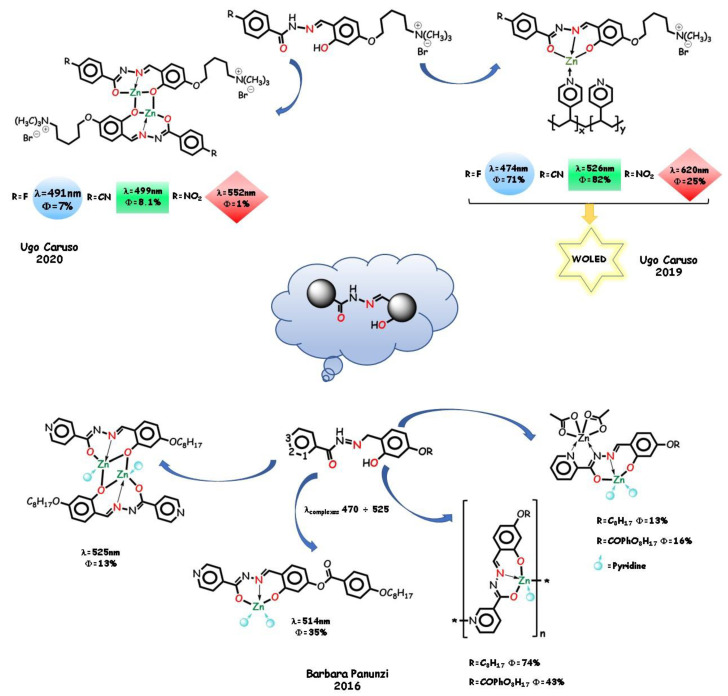
*O*,*N*,*O* aryl-hydrazone ligands with a cationic chain and their zinc polymers. Aroylhydrazone ligands with *orto, meta, para* pyridinoyl moiety.

As a part of the same research, other groups of aroyl- and acylhydrazones were studied for their ability to form stable zinc(II) complexes with a varied coordination environment and tunable photophysical properties. In 2019, U. Caruso and coworkers reported [74,120] on three *O*,*N*,*O* tridentate aryl-hydrazone ligands with a cationic-ended side chain and a different electron-withdrawing substituent (Figure 10). The charged chain makes both ligands and complexes very soluble in common organic solvents and aqueous mixed solvents and emissive in solution, as required in soft-matter solar cells, such as light-emitting electrochemical cells (LECs). RGB (red-green-blue) emission color tuning in ethanol was obtained by increasing the withdrawing strength of the substituent. PLQYs of the complexes are higher with respect to similar zinc coordinated systems [29,74,121,122,123], due to the electrostatic repulsions between the cationic chains and implemented respect to the free ligands, due to the CHEF effect.

The same fluoro, cyano, and nitro substituents and the charged chain guaranteeing solubility were employed by grafting the coordination moieties to a preformed PVPy (Figure 10). The resulting materials show RGB emission tuning in the solid state, with medium to excellent (more than 80% for the green-emissive polymer) PLQYs. By modulating the contents of various emissive pendants into a single polymer chain, in 2020, U. Caruso and coworkers reported a single-component highly performing white emissive material employable in the construction of white OLED devices (WOLED) with CIE coordinates (0.30, 0.31) [74].

In 2016, B. Panunzi and coworkers pointed out the exclusive role of auxiliary pyridine ligands in determining the molecular photophysical properties of the tridentate hydrazine complexes [24] and studied the effect of a pyridine moiety into the main structure of *O*,*N*,*O* aroylhydrazone ligands (Figure 10). Direct involvement of the pyridinoyl moiety in the coordination to the metal was observed when the nitrogen was in the *ortho* or *meta* position. 1-D coordination polymers were obtained with the *meta* derivatives, with 74% PLQY in the solid state. This result suggests that crystalline packed polymeric structures could provide emission enhancement for their continuous rather than discrete structure in the solid state. The tight crystal structure permits an efficient electron hopping.

## 4. Nitrogen, Oxygen, and Sulfur Binding Sites

In several tridentate structures, an oxygen atom was replaced by a, *S* donor binding site. Sulfur–nitrogen chelating agents are employed for their marked biological activities both as ligand and in their transition metal complexes. In many cases, the versatile C=N bond and aromatic heterocycle rings were employed in the ligand construction. Many *N*,*N*,*S* tridentate zinc complexes were explored by paying attention to both structural and spectroscopic behavior. More rarely, *S*,*N*,*S* tridentate pincer ligands with zinc salts [124] were explored, mainly screened for their reactivity and/or catalytic activity rather than for the PL properties. On the other hand, a few significative examples of mixed *N*,*S*,*O* binding sites were recently proposed. In most cases, interest was focused on the X-ray structural exploration of the coordination core and in their basic chemo-physical properties. In some cases, the observation of specific spectroscopic properties promoted the investigation of the emission properties and even moved an applicative interest.

### 4.1. N,N,S Ligands

**Figure 11:**
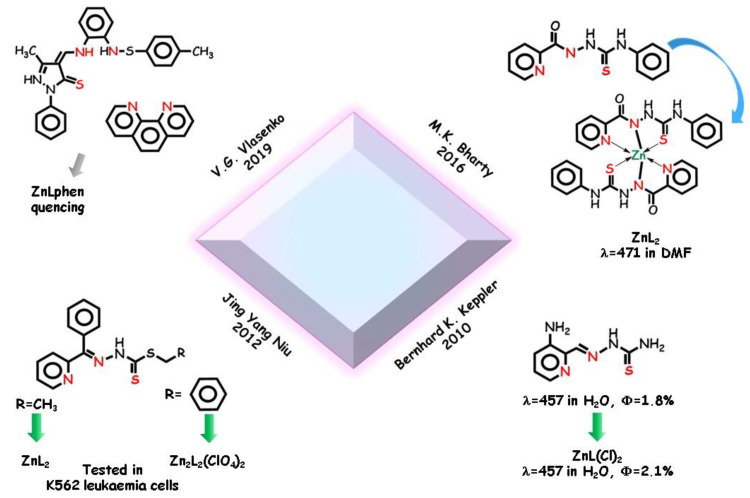
Selected example of *N*,*N*,*S* tridentate pincers and their complexes.

In 2012, Jing Yang Niu and coworkers synthetized two *N*,*N*,*S* tridentate dithiocarbazate-type Schiff base ligands [125] (Figure 11). In the solid state, the ligands are in the thione tautomeric form and the derived mono or dinuclear zinc complexes show different stoichiometry and coordination core. Biological studies showed that the zinc(II) complexes are able to distinguish a leukemia cell line from a normal hepatocyte cell line by a selective fluorescence response. Still, due to their biological interest, thiosemicarbazones and 1,3,4-thiadiazole were employed to build *N*,*N*,*S* ligands by M.K. Bharty and coworkers in 2016 [126] with different metal cations. Zinc acetate was reacted with the fluorescent silent thiosemicarbazide-type ligand and with the derived fluorescent thiadiazole-type ligand producing two zinc complexes with ZnL_2_ stoichiometry, where two negative nitrogen bind the metal. Interestingly, after cyclization, the same ligand acts as an *N*,*N* neutral bidentate ligand toward the zinc cation. The *N*,*N*,*S* tridentate complex is emissive in solution, a phenomenon ascribed to the CHEF effect by formation of four five-membered chelate rings around the cation. By DFT study, the electron density of HOMO in the thiosemicarbazide-type ligand was found on the pyridine ring nitrogen, hydrazinic nitrogen, and thione sulfur. LUMO is localized on the pyridine ring and less on hydrazinic nitrogen and sulfur. The electronic transition from HOMO to LUMO levels are associated with the π,π* transition of ligand.

Thanks to its intrinsic fluorescence properties, triapine ligand (Figure 11) can be used to monitor the uptake and intracellular distribution in cancer cells by fluorescence microscopy. In 2010, Bernhard K. Keppler and coworkers [127] studied the triapine ligand and its tridentate zinc complex. While the compounds show similar emission spectra with a maximum at 457 nm and similar quantum yields in water, distinctly different cellular distributions of the free ligand and its complex were found. In particular, the zinc complex binds with strong affinity to a substructure within the nucleus, providing opportunities in labelling techniques. Very recently, a series of complexes from different transition metal cations were explored by V.G. Vlasenko and coworkers in 2019 [128] (Figure 11). A tridentate *N*,*N*,*S* thioxo-pyrazole Schiff base ligand was employed toward zinc cation with 1,10-phenathroline as auxiliary ligand. In the trigonal bipyramid mononuclear complexes, the amidic and iminic nitrogen atom groups and sulfur thiolate atom group constitute the tridentate site, the coordination sphere being completed by N atoms of phenanthroline. Interestingly, the related zinc complex does not display fluorescence. Computational analysis assigned the experimentally observed bands to π,π* of the tridentate and to π(L), π*(Phen) electronic LLCT transitions.

### 4.2. N,S,O Ligands

**Figure 12:**
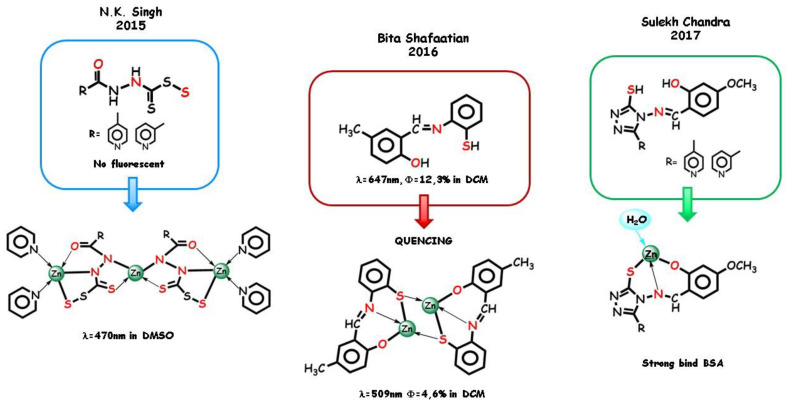
Selected example of *N*,*S*,*O* tridentate pincers and their complexes.

In 2015, N.K. Singh and coworkers synthetized two trinuclear Zn(II) complexes [129] from carboperthioate ligands (Figure 12). In both complexes, the middle zinc cation has a tetrahedral arrangement with two hydrazinic nitrogens and two sulfur atoms from two perthio ligands, structurally similar to the zinc finger protein. Both side zinc cations are five coordinated by one carbonyl oxygen, one hydrazinic nitrogen, and one sulfur from the carboperthioate ligand, which acts as a tridentate pincer toward the side cations. Two of the pyridinic nitrogen atom groups act as auxiliary ligands. Interestingly, from a structural point of view, the trimeric complexes generate self-assembly supramolecular structures in dependence on the different position of the pyridinic nitrogen atom of the ligand. The ligand is fluorescent silent while the complex with the 4-pyridyl substituent displays a blue emission at 470 nm in DMSO, predominantly ascribable to MLCT transitions. In this case, the mobility of the electron transfer in the backbone is enhanced and the electron transition energy of ILCT decreases due to back-coupling of π-bond between the metal and ligand. Moreover, the formation of a five-membered chelate between the coordination units and the central metal ion increases the π,π* conjugation and the conformational coplanarity, consequently decreasing the energy gap between the π and π* molecular orbitals of the ligand.

In 2016, a Schiff base ligand derived from 2-aminothiophenol was coordinated as an *N*,*S*,*O* tridentate ligand to different transition metal cations by Bita Shafaatian and coworkers [19]. The fluorescence properties of the ligand and of the dinuclear complexes (Figure 12) were examined. Interestingly, in all cases, the metal complexes in dichloromethane exhibit weak fluorescence in comparison to ligand. For Zn(II) complex, no emission observed was assigned to π,π* IL transitions. PLQY decreases to about one third with a relevant blue shift in the emission maxima with respect to the free ligand. Finally, as an example of biological application of *N*,*S*,*O* complexes, in 2017, two novel triazole containing Schiff base ligands were employed with zinc cation and other transition metals by Sulekh Chandra and coworkers [130]. The ligands behave as binegative tridentate in the formation of 1:1 aqueous metal complex (Figure 12), which were employed in fluorescence quenching experiments of the strong emission band at 327 nm of BSA, revealing a zinc complex that was more promising due to its strong binding ability.

## 5. Conclusions

One of the biggest challenges of the modern era is sustainability. Over the last years, the high variety of *N*, *O*, *S* donor ligands moved a strong interest toward spectroscopic and applicative features of the related complexes. Tridentate ligands are simple, low cost, and easy synthesizable pincers able to guarantee good stability to the derived complexes. On the other hand, zinc cation has a unique mix of attractive properties, which potentially make it a smart “green” metal. Combinations of suitable tridentate ligands with zinc cation are the perfect union to achieve remarkable PL responses and targeted applications. As a d^10^ closed shell cation, zinc(II) plays a quite innocent role in the electronic and therefore spectroscopic pattern of the ligand, often guaranteeing a relevant CHEF effect. For this reason, zinc is a key issue in developing an alternative class of environmentally friendly and highly efficient fluorophores for display and lighting technologies. On the other hand, zinc plays a crucial role in many important biological processes and is a structural key component of proteins and enzymes. Versatile molecules able in the zinc(II) detection or in the zinc binding of fluorescence markers to specific biological substrates are recently proposed chemo- and biosensors.

As scientists, we presented here some examples of zinc tridentate complexes in an attempt to highlight some of their unique properties. We examined the PL emission tuning due to coordination and stating the most interesting applications. As researchers, we are committed to continuing the study of novel systems for the new technological frontier and we expect our research to be the subject of further interesting discoveries.
